# Use of the knowledge to action model improved physical therapist adherence to a common clinical practice guideline across multiple settings: a multisite case series

**DOI:** 10.1186/s12913-022-08796-4

**Published:** 2022-12-01

**Authors:** Julie K. Tilson, Clarisa A. Martinez, Sara MacDowell, Linda J. D’Silva, Robbin Howard, Heidi R. Roth, Karen M. Skop, Elizabeth Dannenbaum, Lisa Farrell

**Affiliations:** 1 Division of Biokinesiology and Physical Therapy, University of Southern California, Los Angeles, California USA; 2 Physical Therapy, Hearing and Balance Center, Our Lady of the Lake Regional Medical Center, Baton Rouge, Louisiana USA; 3 Physical Therapy, Rehabilitation Science, and Athletic Training, University of Kansas Medical Center, Kansas City, Kansas USA; 4 Northwestern University School of Physical Therapy and Human Movement Sciences and Shirley Ryan AbilityLab, Chicago, IL USA; 5 Physical Medicine and Rehabilitation Services, Department of Physical Therapy, James A. Haley Veterans’ Hospital, Morsani College of Medicine, University of South Florida, School of Physical Therapy, Tampa, FL USA; 6 Vestibular Program, Jewish Rehabilitation Hospital Foundation, Laval, Quebec Canada; 7 Symmetry Alliance, LLC, Fort Lauderdale, Florida USA

**Keywords:** Clinical practice guideline, Implementation, Knowledge translation, Physical therapy, Vestibular rehabilitation, Case series, Knowledge to action model, Audit and feedback, Reminders, Communities of practice

## Abstract

**Background:**

When a new guideline is published  there is a need to understand how its recommendations can best be implemented in real-world practice. Yet, guidelines are often published with little to no roadmap for organizations to follow to promote adherence to their recommendations. The purpose of this study was to evaluate the impact of using a common process model to implement a single clinical practice guideline across multiple physical therapy clinical settings.

**Methods:**

Five organizationally distinct sites with physical therapy services for patients with peripheral vestibular hypofunction participated. The Knowledge to Action model served as the foundation for implementation of a newly published guideline. Site leaders conducted preliminary gap surveys and face-to-face meetings to guide physical therapist stakeholders’ identification of target-behaviors for improved guideline adherence. A 6-month multimodal implementation intervention included local opinion leaders, audit and feedback, fatigue-resistant reminders, and communities of practice. Therapist adherence to target-behaviors for the 6 months before and after the intervention was the primary outcome for behavior change.

**Results:**

Therapist participants at all sites indicated readiness for change and commitment to the project. Four sites with more experienced therapists selected similar target behaviors while the fifth, with more inexperienced therapists, identified different goals. Adherence to target behaviors was mixed. Among four sites with similar target behaviors, three had multiple areas of statistically significantly improved adherence and one site had limited improvement. Success was most common with behaviors related to documentation and offering patients low technology resources to support home exercise. A fifth site showed a trend toward improved therapist self-efficacy and therapist behavior change in one provider location.

**Conclusions:**

The Knowledge to Action model provided a common process model for sites with diverse structures and needs to implement a guideline in practice. Multimodal, active interventions, with a focus on auditing adherence to therapist-selected target behaviors, feedback in collaborative monthly meetings, fatigue-resistant reminders, and developing communities of practice was associated with long-term improvement in adherence. Local rather than external opinion leaders, therapist availability for community building meetings, and rate of provider turnover likely impacted success in this model.

**Trial registration:**

This study does not report the results of a health care intervention on human participants.

**Supplementary Information:**

The online version contains supplementary material available at 10.1186/s12913-022-08796-4.

## Contributions to the literature

- This multi-site case series describes use of the Knowledge to Action model to guide implementation of provider-selected target behaviors from a clinical practice guideline.
- Key features of site-selected multi-modal interventions to change therapist behavior included audit and feedback, fatigue-resistant reminders, and communities of practice.
- Local opinion leaders, therapist availability for community building meetings, and low provider turnover may be important elements to success.
- Therapist demographics, specifically, years of specialty practice experience, may impact target behaviors selected for implementation.

## Background

When a new guideline is published there is a need to understand how its recommendations can best be implemented in real-world practice. Timely and effective implementation of new guidelines has the potential to optimize the quality of care delivered to patients and to reduce unwarranted variation in care [1]. Effective mechanisms for guideline implementation are emerging, and have proven to be heterogenous [2–5]. Furthermore, for physical therapy practice, which often involves multi-component, complex interventions, evidence for effective guideline implementation across multiple settings is limited [6–8].

Peripheral vestibular hypofunction is a condition that results from reduced neurologic input from one or both vestibular organs in the inner ear. It is estimated that one third of US adults over the age of 40 experience vestibular dysfunction [9] which can result in disabling symptoms including dizziness, vertigo, blurred vision during head movement, postural instability, fear of movement, anxiety, and depression. Vestibular rehabilitation includes identification of the nature of a patient’s vestibular dysfunction and prescription of exercise-focused interventions to promote gaze stability and habituation. Patients must complete gaze stabilization exercises daily for best outcomes [10, 11].

The guideline, “Vestibular Rehabilitation for Peripheral Vestibular Hypofunction: An Evidence-Based Guideline” was published in 2016 [10] by the Academy of Neurologic Physical Therapy. The guideline contains ten action statements describing evidence-based practice for management of vestibular hypofunction, including: who should be offered vestibular rehabilitation, which outcome measures to utilize, specific strategies for exercise prescription and dosing, when care might be impacted by comorbidities, and when to discontinue care. The guideline was rated high quality [12] by the American Physical Therapy Association using the Appraisal of Guidelines, Research and Evaluation Instrument II (AGREE II) [13]. A taskforce of clinician and implementation leaders from the United States and Canada was formed in 2016 by the Academy of Neurologic Physical Therapy to facilitate implementation of the recently published peripheral vestibular hypofunction guideline [10].

The pragmatic nature of implementation studies necessitates the use of conceptual frameworks and models to support comprehensive design for multi-component interventions and interpretation of the contextual factors that impact their outcomes [7, 14, 15]. Additionally, evidence suggests that active, multi-component knowledge translation interventions can be effective to enhance knowledge and practice behaviors of physical therapists [2, 16, 17]. Among many mechanisms for supporting implementation defined by the Cochrane Effective Practice and Organization of Care (EPOC) taxonomy [18], audit and feedback regarding clinician performance [4, 19–21], reminders designed to limit alert fatigue [4, 22], and internal leadership to faciltiate communities of practice [4, 23, 24] are likely to be effective for promoting clinician behavior change to align with guideline recommendations.

The purpose of this case series was to evaluate the impact of using a common procedural model to implement a single clinical practice guideline across multiple physical therapy clinical settings. We provide standardized, detailed descriptions [25] of each site’s implementation process and interventions to support replication and iteration by others.

## Methods

### Case descriptions

#### Setting and participants

Five sites with rehabilitation services for patients with peripheral vestibular hypofunction participated in this organizational case series (Table 1). Opinion leaders, from the Academy of Neurologic Physical Therapy Peripheral Vestibular Hypofunction Guideline Dissemination and Implementation Taskforce, served as site leaders for the project. Four site leaders were practicing physical therapists at the participating sites (local opinion leaders), one site (site C) was led by a collaborating academic physical therapist. All sites agreed to participate and secured institutional review board approval. Site leaders and the principal investigator met twice per month from project inception through the completion of data collection to facilitate collaboration and project fidelity across sites.

**Table 1:** Site Characteristics

Site/Location	Type of Organization	Site Leader: Time at Facility (years)	Site Leader: Practice Experience with Peripheral Vestibular Hypofunction (years)	Number of Participating Clinic Locations	Target Guideline Action Statements Selected by Therapist Participants
Site A Chicago, IL	Non-profit teaching hospital	16	9	1	5 and 7
Site B Baton Rouge, LA	Private, non-profit	2	9	1	5 and 7
Site C Kansas City, MO	Private, for profit	N/A	15	3	5, 7, and 9
Site D Los Angeles, CA	Academic Medical Center	15	11	1	5, 7, and 9
Site E Tampa, FL	U.S. Government	9	19	9	1, 2, 3, and 9

Relevant Action Statements From the guideline [10]:

Action Statement 1: EFFECTIVENESS OF VESTIBULAR REHABILITATION IN PERSONS WITH ACUTE AND SUBACUTE UNILATERAL VESTIBULAR HYPOFUNCTION. Clinicians should offer vestibular rehabilitation to patients with acute or subacute unilateral vestibular hypofunction. (Evidence quality: I; recommendation strength: strong)

Action Statement 2: EFFECTIVENESS OF VESTIBULAR REHABILITATION IN PERSONS WITH CHRONIC UNILATERAL VESTIBULAR HYPOFUNCTION. Clinicians should offer vestibular rehabilitation to patients with chronic unilateral vestibular hypofunction. (Evidence quality: I; recommendation strength: strong)

Action Statement 3: EFFECTIVENESS OF VESTIBULAR REHABILITATION IN PERSONS WITH BILATERAL VESTIBULAR HYPOFUNCTION. Clinicians should offer vestibular rehabilitation to patients with bilateral vestibular hypofunction. (Evidence quality: I; recommendation strength: strong)

Action Statement 5: EFFECTIVENESS OF DIFFERENT TYPES OF EXERCISES IN PERSONS WITH ACUTE OR CHRONIC UNILATERAL VESTIBULAR HYPOFUNCTION. Clinicians may provide targeted exercise techniques to accomplish specific goals appropriate to address identified impairments and functional limitations. (Evidence quality: II; recommendation strength: moderate)

Action Statement 7: OPTIMAL EXERCISE DOSE OF TREATMENT IN PEOPLE WITH PERIPHERAL VESTIBULAR HYPOFUNCTION (UNILATERAL AND BILATERAL). Clinicians may prescribe a home exercise program of gaze stability exercises consisting of a minimum of 3 times per day for a total of at least 12 minutes per day for patients with acute/subacute vestibular hypofunction and at least 20 minutes per day for patients with chronic vestibular hypofunction. (Evidence quality: V; recommendation strength: expert opinion)

Action Statement 9: FACTORS THAT MODIFY REHABILITATION OUTCOMES. Clinicians may evaluate factors that could modify rehabilitation outcomes. (Evidence quality: I-III; recommendation strength: weak to strong)

#### Implementation intervention using a common model

**Fig. 1:**
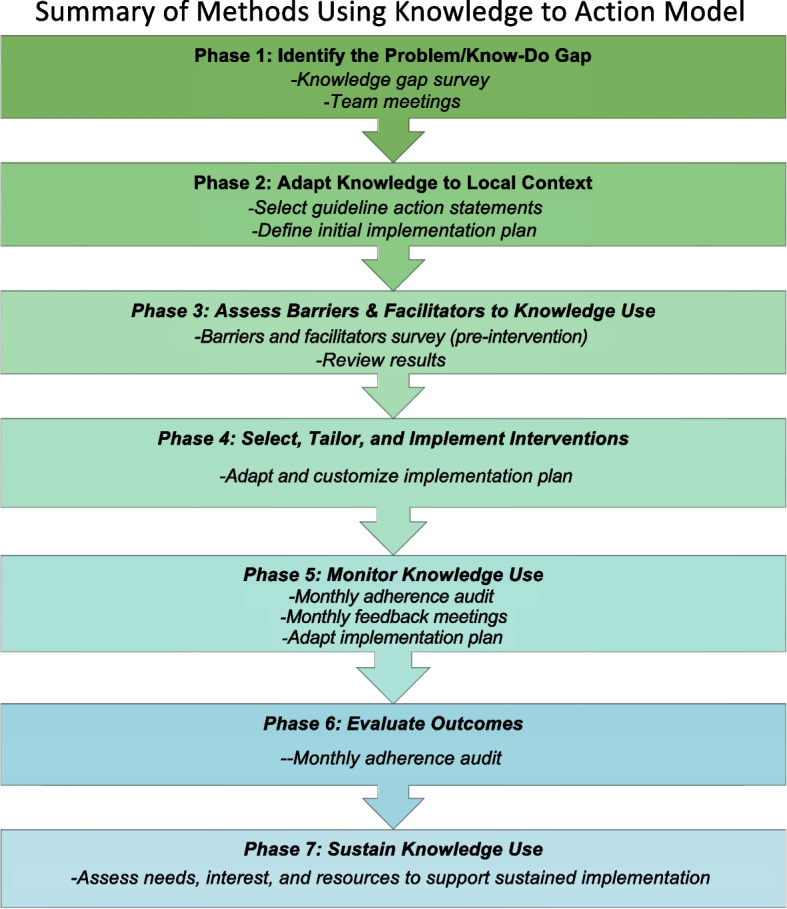
Summary of methods for each phase of the outer circle (‘action cycle’) of the Knowledge to Action model [26]

The Knowledge to Action process model [26] served as the foundation for implementation at each site. The seven phases of the outer circle (‘action cycle’) of the Knowledge to Action model are outlined in Fig. 1. The methods that follow are organized by these phases.

##### Phase 1: identify the problem/know-do gap

Site leaders conducted preliminary gap surveys (Supplementary File 1) to assess therapist practice patterns and knowledge of the guideline. Surveys were followed by face-to-face meetings with therapist and administrative stakeholders to present the peripheral vestibular hypofunction guideline action statements, including a single page summary education document [27], and stakeholder survey results. Each site’s stakeholder group was asked to answer the following question: “Based on the ten action statements from the guideline, is there something that we would like to change in our practice to improve the care we provide to patients?” This question, designed to identify each sites’ ‘knowledge-practice gap’, was addressed over multiple meetings at each site.

##### Phase 2: adapt knowledge to the local context

Each site’s stakeholders engaged in a local consensus process whereby they identified the action statements that they would focus on for the duration of the study. Thus, the ‘problem’ identified at each site was the opportunity to improve adherence to specific guideline action statements.

Site leaders and therapists met to create evidence-based implementation interventions to improve therapist adherence to their selected action statements (Table 1). Barriers to adherence were discussed and potential implementation strategies proposed. Each site leader used therapist feedback to define an intervention plan for their site.

##### Phase 3: assess barriers/facilitators to knowledge use

A standardized survey was sent to each participating therapist to formally assess perceived barriers and facilitators to the implementation plan, and organizational readiness for change. The survey assessed participating therapists’ clinical demographics; the Organizational Readiness to Implement Change (ORIC) [28] (10-item, 5-point likert scale assessment with established psychometric performance for reliability and validity for assessing change commitment and change efficacy); and fourteen 5-point likert scale items adapted from the Consolidated Framework for Implementation Research (CFIR) Index Manual 3.0 [29] related to four dimensions that impact implementation: Intervention Characteristics, Outer Setting, Inner Setting, and Characteristics of Individuals [3]. Items with < 65% of respondents answering either agree or strongly agree for positive feelings about the intervention were identified as potential barriers. The study principal investigator met with each site leader to review site-specific results of the survey as they adapted their plan for implementation.

##### Phase 4: select, tailor, and implement interventions

Site leaders led design of a six-month, staged, adaptive, active, and multi-modal implementation plan, customized to the needs, culture, and context of their site. Implementation interventions designed to impact therapist behavior change are summarized in Table 2 and described in detail in Supplementary File 2 using the WIDER checklist for reporting of knowledge translation interventions [25]. Therapist-selected target behaviors to improve adherence to the guideline are summarized in Table 3 and described in detail in Supplementary File 3.

**Table 2:** Implementation Intervention Strategies

Strategy	EPOC Definition	***Example*** Intervention	A	B	C	D	E
Audit and Feedback	A summary of health workers’ performance over a specified period of time, given to them in a written, electronic or verbal format. The summary may include recommendations for clinical action.	Monthly chart reviews to assess therapist adherence to target clinical behaviors.	✔	✔	✔	✔	✔
Communities of Practice	Groups of people with a common interest who deepen their knowledge and expertise in this area by interacting on an ongoing basis.	Monthly meetings amongst participating therapists to discuss practice for target patient population.	✔	✔	✔	✔	✔
Educational Materials	Distribution to individuals, or groups, of educational materials to support clinical care.	Handouts summarizing clinical practice guideline.	✔	✔	✔	✔	✔
Educational Meetings	Courses, workshops, conferences, or other educational meetings.	Educational program with specific learning objectives and tasks	✔				✔
Local Consensus Process	Formal or informal local consensus processes.	Identification of specific target behaviors to facilitate adherence to guideline recommendations.	✔	✔	✔	✔	✔
Local Opinion Leaders	The identification and use of identifiable local opinion leaders to promote good clinical practice.	Site leaders serving as resources for therapists, promoting best practice	✔	✔		✔	✔
Reminders	Manual or computerised interventions that prompt health workers to perform an action during a consultation with a patient.	Digital tools integrated into documentation system to remind therapists of questions to ask patients and information to record in medical record.	✔	✔	✔	✔	
Resources provided to therapists to offer to patients	N/A	Patient educational and/or exercise instruction handouts.	✔	✔	✔	✔	✔

*Definitions informed by Effective Practice and Organisation of Care EPOC Taxonomy, 2015 [18]

**Table 3:** Therapist Target Behaviors

**Expectation**	**Therapist Target Behavior**	**Site A**	**Site B**	**Site C**	**Site D**	**Site E**
Offer Resource to Patient During Episode of Care	Educational Handout(s)	✔		✔		
	Exercise Instruction Handout		✔		✔	
	Exercise Instruction Videos	✔				
	Exercise Log Handout		✔			
	Timers		✔	✔		
	Metronome			✔		
	GS Exercise Targets		✔			
	Text Message/Smart Phone Communication Tool	✔	✔	✔	✔	
Record Each Visit	GS Exercises Practiced with Patient				✔	
	GS Exercise Program Prescribed/Advanced	✔	✔	✔	✔	
	Prescribed GS Dose Recorded	✔	✔	✔	✔	
	Inquired About Patient GS Exercise Adherence	✔	✔	✔	✔	
	Inquired About Patient’s Daily Minutes of GS Exercise	✔	✔			
Complete During Episode of Care	Provide Referral Resource if Symptoms Consistent with Anxiety			✔		
	Screen for Symptoms of Depression				✔	
	Screen for Symptoms of Anxiety				✔	
	Assess Patients for Vestibular Dysfunction					✔
Therapist Training Program for Assessing Vestibular Dysfunction	Complete in-person Education Course					✔
	Demonstrate Competency for Assessing Peripheral Vestibular Hypofunction					✔

Details for each intervention are provided in Supplementary File 3

GS: Gaze Stabilization - the specific type of exercises recommended by the guideline for patients with peripheral vestibular hypofunction

##### Phase 5: monitor knowledge use

All sites conducted monthly audits of therapist adherence to target behaviors. Monthly feedback meetings were held each month to provide therapists with detailed feedback about group adherence. Feedback reports were followed by discussion about facilitators and barriers to adherence, therapist education, and group problem-solving to optimize adherence in the coming month. Implementation interventions were revised and adapted to optimize adherence as needed. Sites A-D monitored adherence through chart review. Site E monitored adherence through tallies of how many patients were assessed for peripheral vestibular hypofunction within each participating location.

##### Phase 6: evaluate outcomes

At the conclusion of each site’s implementation intervention, the site leads for sites A-D conducted an electronic medical record review of their sites’ therapists’ adherence to site-selected goals for the 6 months prior to starting the implementation study and the 6 months following the implementation phase. Chart review data included descriptive patient characteristics and participant adherence to site-selected goals. Adherence was determined by presence/absence of chart documentation related to the goal, for instance, “Did the therapist document exercise dose for vestibular exercises? (Yes/No)”. Adherence greater than 75% of episodes of care or visits was considered strong, change in adherence greater than 50 percentage points was considered large.

Therapists at site E completed a standardized self-efficacy survey [30] adapted to caring for individuals with peripheral vestibular hypofunction at the start and end of the six-month intervention. Site E also tracked therapist participation in training and competency assessment activities, and the total number of patients per month assessed for peripheral vestibular hypofunction.

##### Phase 7: sustain knowledge use

Each site determined a plan to continue to promote adherence to the target therapist behaviors after the six-month intervention. These were carried into the six-month post intervention phase. At the conclusion of the intervention period, therapists were asked to complete the barriers and facilitators survey a second time.

#### Data collection and analysis

The primary outcome for the case series analysis was difference in adherence to site-specific goals before and after the implementation intervention. Chart review, tally counts, and survey data were collected using REDCap electronic data capture tools hosted at the University of Southern California and reported using descriptive statistics.

Chart reviews (collected for the 6 months before and after the intervention) included patients that: 1) were seen for an initial evaluation during the 6-month time period, and 2) had a diagnosis of peripheral vestibular hypofunction. After identifying appropriate charts to include in the pre or post sample, the medical record was reviewed. Each chart reviewer looked at treating therapist documentation to determine compliance at two levels: 1) adherence across the whole episode of care (defined as the time between initial evaluation and discharge) and 2) adherence at each patient encounter. For example, offering a patient the opportunity to sign up for reminder text messages was expected to occur at one time point across an episode of care, while recording the dose of a patient’s prescribed home exercises was expected to occur at each patient encounter. Chart reviewers also established criteria at their site to determine if a compliance behavior was not applicable to a given patient encounter. For example, a patient without symptoms consistent with anxiety would be excluded from a count for therapist behaviors that should be provided to patients with those symptoms. Patient visits in which the compliance question was not applicable were excluded from that analysis. As a result, the total number of patient records assessed for episode of care level target behaviors and patient visit level target behaviors varied.

Because of the case series nature of the study, separate analyses were conducted for each site to determine change in adherence using chi-square and t-test to measure differences in pre- to post-goal adherence. We used a Bonferroni adjustment based on the number of variables tested per site to mitigate spurious findings due to multiple comparisons. All analyses were conducted using SAS Education edition statistical software.

## Results

Therapist participant characteristics by site are described in Table 4. Results are reported by site due to the case series nature of the study design and analysis.

**Table 4:** Therapist Participant Demographics

Therapist characteristics					Therapist report of numbers of patients with PVH/month		
Site	Participants (start of study)	Mean years since graduation (SD)	Mean years treating patients with PVH (SD)	% with advanced training	0–10 PVH patients	11–20 PVH patients	> 20 PVH patients
A	11	10.4 (6.4)	6.4 (4.5)	11 (100%)	6 (54.5%)	3 (27.3%)	2 (18.2%)
B	2	6.0 (5.7)	5.0 (4.2)	2 (100%)	0 (0%)	0 (0%)	2 (100%)
C	4	14 (9.2)	4.5 (4.8)	3 (75%)	0 (0%)	0 (0%)	4 (100%)
D	4	8 (7.3)	6.6 (5.5)	4 (100%)	3 (75%)	1 (25%)	0 (0%)
E	22	8.4 (8.9)	3.4 (5.2)	14 (63.6%)	17 (77.3%)	1 (4.5%)	4 (18.2%)

*SD* Standard Deviation, *PVH* Peripheral Vestibular Hypofunction

**Table 5:** Site E Implementation Program Results

Implementation Goal	Result
**In-Person Education Course**	
Participants (% of invited population of therapists)	17 Participated (81%)
**Competency Assessment**	
Participants % Achieved Competency	21 Participated 100% Achieved Competency
**Change in Therapist Self Efficacy for Assessing and Treating Patients with PVH**	
Pre-Self-Efficacy Median Score (*n* = 20)	41/65
Post-Self-Efficacy Median Score (*n* = 10)	56.5/65
Absolute Change	15.5

### Site A

**Fig. 2:**
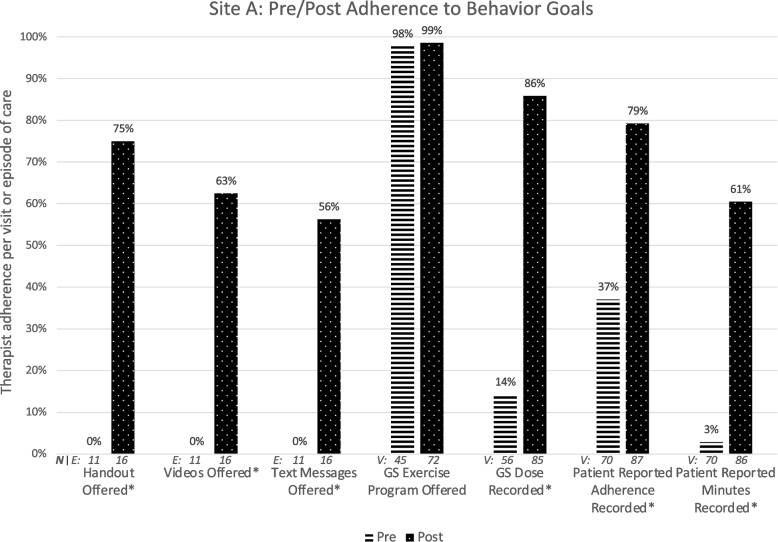
Change in adherence to seven goal behaviors targeted for implementation at Site A. ‘*’ indicates statistically significant change; E indicates variables measured once for episode of care; V indicates variables measured at each visit; GS: Gaze stabilization

Figure 2 illustrates Site A therapists’ target behavior adherence pre and post intervention.

The barriers and facilitators survey (CFIR-informed questions and all ORIC items) was completed by 11 therapists before and 8 therapists after the intervention (100 and 67% of those invited). Of 24 items, three items fell below 65% positive agreement at the pre or post survey [CFIR-informed item: It will be difficult to fit this intervention into our existing workflow (disagree/strongly disagree: pre = 91%/post = 57%); ORIC: People who work here will do whatever it takes to implement this change (agree/strongly agree: pre = 55%/post = 63%); People who work here feel confident that they can manage the politics of implementing this change (agree/strongly agree: pre = 64%; post = 63%)]. Barrier and facilitator survey responses for all sites are available in Supplementary File 4.

### Site B

**Fig. 3:**
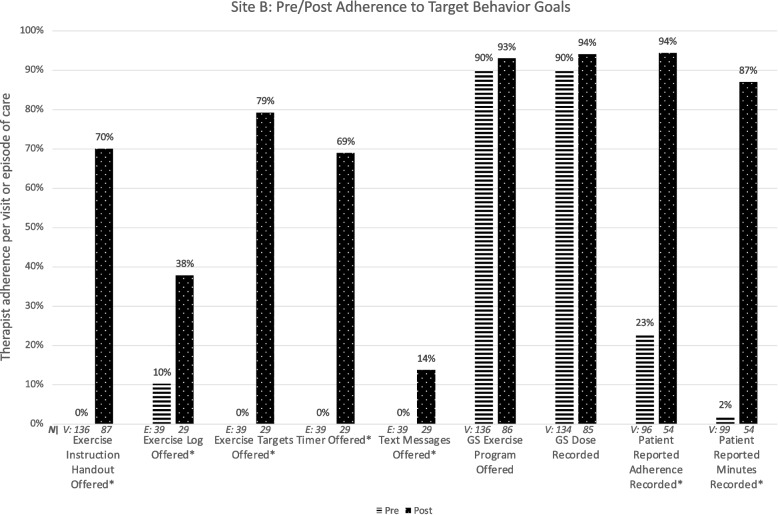
Change in adherence to nine specific goal behaviors targeted for implementation at Site B. ‘*’ indicates statistically significant change. HEP: Home exercise program; GS: Gaze stabilization

Figure 3 illustrates Site B therapists’ target-behavior adherence pre and post intervention. The target behavior ‘Text Messages Offered’ was purposely discontinued due to excessive staff burden and low patient interest during the post intervention phase.

The barriers and facilitators survey (CFIR-informed questions and all ORIC items) was completed by 2 therapists (100% of those eligible) before and after the intervention. Of 24 items, none fell below 65% positive agreement at the pre or post survey.

### Site C

**Fig. 4:**
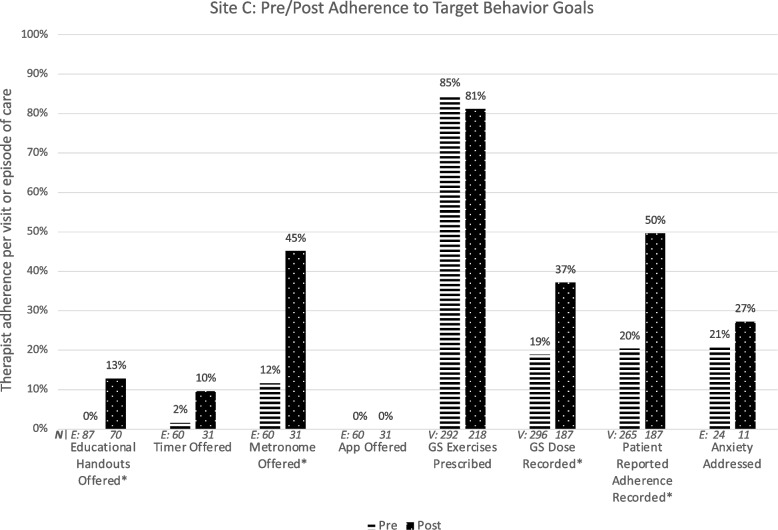
Change in adherence to eight specific goal behaviors targeted for implementation at Site C. ‘*’ indicates statistically significant change. HEP: Home exercise program; GS: Gaze stabilization

Figure 4 illustrates Site C therapists’ target behavior adherence pre and post intervention. The target behavior ‘App Offered’ was ultimately not implemented due to lack of therapist interest.

The barriers and facilitators survey (CFIR-informed questions and all ORIC items) was completed by 4 therapist before and 3 therapists after the intervention (57 and 38% of those eligible). Of 24 items, one fell below 65% positive agreement at the pre or post survey [CFIR-informed item: There is a strong need for this intervention at our facility (pre: 50%; post 33%)].

### Site D

**Fig. 5:**
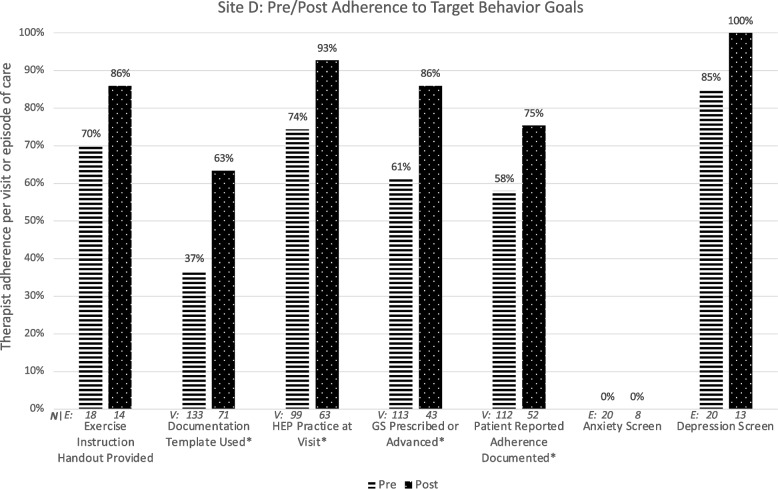
Change in adherence to six specific goal behaviors targeted for implementation at Site D. ‘*’ indicates statistically significant change. HEP: Home exercise program

Figure 5 illustrates Site D therapists’ target behavior adherence pre and post intervention.

The barriers and facilitators survey (CFIR-informed questions and all ORIC items) was completed by 4 therapists before and 3 therapists after the intervention (100 and 75% of those eligible). Of 24 items, three items fell below 65% positive agreement at the pre or post survey [CFIR-informed item: This intervention is in alignment with external incentives and pressures our facility is dealing with (pre: 50%/post: 67%); I have the information I need to implement this intervention (pre: 100%/post: 33%); ORIC: People who work here are determined to implement this change (pre: 75%/post: 33%)].

### Site E

**Fig. 6:**
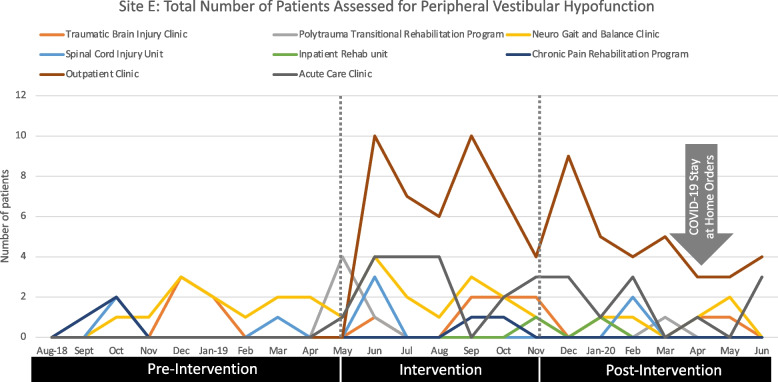
Frequency of patients assessed for peripheral vestibular hypofunction at each of eight service areas for Site E. Post intervention phase was likely impacted by stay-at-home orders issued in this site’s state on April 1, 2020

Site E used participation and self-efficacy criteria to assess their implementation strategy. Table 5 outlines participation results for therapists invited to participate in an in-person education course and those who successfully completed a competency assessment as well as pre and post self-efficacy scores for participating therapists. Figure 6 illustrates the rate of screenings for peripheral vestibular hypofunction conducted by therapists at settings that did not specialize in vestibular rehabilitation.

The barriers and facilitators survey was completed by 22 before and 17 therapists after the intervention (100 and 77% of those eligible). Of 24 items, two fell below 65% positive agreement at the pre or post survey [CFIR-informed item: This intervention is in alignment with external incentives and pressures our facility is dealing with (pre: 64%/post: 93%); ORIC: People who work here feel confident that they can handle the challenges that might arise in implementing this change (pre: 86%/post: 35%)].

## Discussion

We evaluated the impact of using the Knowledge to Action model (Graham 2006) to implement multicomponent interventions to enhance physical therapist adherence to a single clinical practice guideline across multiple clinical settings. Change in adherence to target therapist behaviors was mixed, some sites had consistent, substantial improvements while others had limited observable change in therapist behavior. Here we compare and contrast implementation strategies and outcomes across different clinical environments using the same process model to implement a common guideline.

### Common strategies across sites

The Knowledge to Action model is process model that guides the multi-dimensional translation process [31]. This model provided procedural structure for our sites to design, implement, monitor, and sustain knowledge translation efforts over extended periods of time and through changes in personnel and competing contextual demands. While used extensively in knowledge translation, few rehabilitation studies to date have assessed the use of the knowledge to action model for implementing a singular guideline [7]. Use of all seven phases of the model’s action cycle may be important for achieving effective behavior change. Similar studies to ours without an emphasis on monitoring of behavior change showed limited long-term benefits despite comprehensive, multi-modal, and active interventions [32–34].

Among the components of our sites’ mulit-modal implementation interventions, we propose that audit and feedback was an essential ingredient for success. Our observations are consistent with reports that audit and feedback facilitates clinician behavior change by helping to change social systems and behavior norms within the clinical setting [4]. Our site leaders provided therapists with regular audit and feedback over a sustained period with clear targets. Additionally, many target behaviors had low rates of adherence initially. These characteristics matched essential criteria for effective audit and feedback interventions identified by Ivers and colleagues [19]. Additionally, the collaborative meetings where feedback was given matched Hysong and colleagues [20, 21] findings that audit and feedback improved effectiveness when delivered regularly (at least monthly), in a non-punitive manner, and with specific suggestions for improvement.

The critical nature of audit and feedback for providing practitioners with actionable data for implementing guidelines has been identified in rehabilitation care for individuals with stroke and congenital muscular torticollis [35–38]. It is important to note that audit and feedback can involve substantial administrative burden [23]. Successful broad-scale implementation of this strategy would be enhanced by automated mechanisms for data collection and analysis.

Establishing communities of practice was likely also important to our sites’ successes. Site therapists came to consensus at critical junctures in the study through collegial, regular interactions, under the direction of an opinion leader. High impact facilitators and strong team communication were identified by therapists and nurses as important for implementing best practice recommendations in rehabilitation centers across Canada [24]. Likewise, health care aides working in long-term care reported that having a peer lead their implementation efforts was a logical extension of the team-based nature of their work place [23]. Anecdotal evidence from our study suggests that rare instances of therapist stakeholders not feeling included in a site’s community of practice could deterred implementation.

### Common findings among sites

All target behaviors aimed at improving therapists’ documentation showed statistically significant improvement. In addition to audit and feedback, reminders, may have facilitated this success. While inherently complex computerized decision support systems and associated reminder fatigue have been studied extensively in hospital-based practice [39–42], our sites’ therapists developed simple reminder templates, integrated into their medical record systems, to facilitate target behaviors for documentation. Though different for each site, the reminder templates were consistent with recommendations for reducing alert fatigue including: simple action items, flexibility integrated into workflow, and developed to meet goals valued by the providers [22]. The capacity for physical therapists to make substantial improvements in documentation to better adhere to specific guideline recommendations has been identified by others [43] and may be a fruitful target area for clinical facilities taking their first steps into implementation efforts.

Two areas of implementation were broadly unsuccessful across sites. The first was having therapists offer electronic reminder services (text messages or mobile application communications) to provide daily exercise reminders. Therapists had originally expressed enthusiasm about these tools based on a study of similar tools for patients with musculoskeletal conditions [44]. However, all sites that planned or implemented a version of these services found that the technology was burdensome to set up and maintain and imposed a challenging financial burden. Despite substantial effort to initiate these services at three sites, none continued as of 1 year after the intervention.

The second area that was not broadly successful, was the implementation of screening and support systems for patients experiencing anxiety associated with vestibular hypofunction. Others have shown similar challenges, with as low as 0% compliance in anxiety and depression screening over time amongst speech therapists [36]. We suspect that the anxiety screening and intervention efforts were not sufficiently built into the therapists’ workflow. As others have proposed, therapists may not have been comfortable, or felt sufficiently trained to address anxiety [36]. Interestingly, our findings suggest that high adherence to these types of behaviors, specifically screening for depression, can be achieved.

### Disparate findings among sites

Three sites included distribution of low technology resources to patients as target behaviors to promote patients’ exercise compliance. Two sites had consistent success with these efforts. One site had some success, but lower compliance overall with similar target behavior goals. We suspect that this was related to four factors. First, the site with more challenges had a small number of therapists spread over multiple facilities. The combination of geographic distance and staggered break schedules made it difficult to schedule monthly meetings where all could attend. This likely reduced the effectiveness of feedback and stunted the development of an effective community of practice. Second, the site experienced a high rate of therapist turnover including the addition of a third physical location during the study. Third, this site was the only site using an external site lead. Evidence suggests that while external facilitators can play an important role in implementation, strong internal facilitators are needed for ultimate success [45].

While four sites of different size and organizational structure chose similar implementation goals, our largest site, a US Veterans’ Administration facility, had distinctly different needs. This site had the largest number of therapists with the least experience treating patients with peripheral vestibular dysfunction. Therapists at the site determined that rather than focusing on exercise prescription and adherence for therapists treating the target population, they wanted to address the need for therapists across their service areas to assess, identify, and initiate services for patients with previously undiagnosed peripheral vestibular hypofunction. Thus, the site developed a comprehensive training and resource program for their therapists. Use of comprehensive educational efforts to increase patient access to guideline-informed care may also be driven by cultural values and objectives in the US Veteran’s Administration. Similar implementation approaches have been reported for veterans with lower limb amputations [46], chronic pain [47], spinal cord injury [48]. It was difficult to thoroughly analyze the impact of our intervention at this site because the post intervention phase was impacted by stay-at-home orders caused by the COVID-19 pandemic. Fortunately, this was the only site still collecting data with then pandemic began.

### Limitations

There are several important limitations to consider when interpreting the results of this study. First, this was a case series by design, with the purpose of allowing each site to apply the knowledge to action model to their unique context and site needs. Thus, we did not aggregate findings across sites. Second, we used a pre post analysis to determine impact. Thus, we cannot definitively determine that our interventions caused the changes observed. However, no other interventions were targeting the measured therapist behaviors and common consistencies between sites suggests a likelihood that the implementation interventions directly impacted the changes observed. Third, the number of therapist-patient encounters were sufficiently small to justify caution in extrapolation to other facilities. The similarity of findings between sites with similar goals and strategies suggests potential for comparable results beyond our study. Finally, the complex and pragmatic nature of the sites’ implementation efforts makes it difficult to quantify and characterize all of the potential factors influencing study findings.

## Conclusions

The Knowledge to Action model [12] provided a common framework for sites with diverse structures and needs to implement a common guideline in practice. Multi-modal, active interventions, with a focus on auditing adherence to therapist-selected target behaviors, feedback in collaborative monthly meetings, fatigue-resistant reminders, and communities of practice was associated with meaningful long-term improvement in adherence. Success was most common with behaviors related to documenting prescribed exercises, collecting and documenting patient reports about home exercise adherence, and offering patients low technology resources. The least success was experienced with offering patients high technology resources and addressing patients with symptoms consistent with anxiety. Local opinion leaders, therapist availability for community building meetings, and low rate of provider turnover may be important elements to success. With those considerations in mind, we recommend use of the knowledge to action model to guide multimodal interventions that include regular audit and feedback in communities of practice to implement therapist-selected target behaviors to enhance adherence to newly published clinical practice guidelines.

## Supplementary Information

**Additional file 1.**

## Data Availability

The datasets used and/or analyzed during the current study are available from the corresponding author on reasonable request.

## Abbreviations

CFIR: Consolidated Framework for Implementation Research
GS: Gaze Stabilization
HEP: Home Exercise Program
ORIC: Organizational Readiness for Implementing Change
PVH: Peripheral Vestibular Hypofunction
SMS: Short Message Service
