# Supplementary material for: Use of the knowledge to action model improved physical therapist adherence to a common clinical practice guideline across multiple settings: a multisite case series
Source: BMC Health Serv Res. 2022 Dec 1;22:1462. doi: 10.1186/s12913-022-08796-4 (PMC9714412; doi:10.1186/s12913-022-08796-4)
Supplement: Supplementary file 1 — Additional file 1. [file 12913_2022_8796_MOESM1_ESM.docx]

**Supplementary File 1**

**Gap Analysis Survey**

Q1 Please identify the facility you are associated with:

Q2 How many years have you been protecting as a PT

0-5

6-10

11-15

16-20

20+

Q3. For how many years have regularly cared for patients with vestibular hypofunction?

0-2

3-5

6-10

11-15

16-20

20+

Q4. Approximately how many treatment sessions per month do you care for persons with vestibular hypofunction?

0-2

3-5

6-10

11-15

16-20

20+

Q5. Which of the following do you believe are likely to impact patients prognosis for recovery? Select all that apply

age

gender

delayed start to rehabilitation

Comorbidities (anxiety, migraine, peripheral neuropathy)

Use of vestibular suppressants

Q6. Do you see vestibular hypofunction patients predominantly in an inpatient or outpatient setting?

Mostly inpatient

mostly outpatient

Even split between the two

Q7. How familiar are you with this clinical practice guideline: Hall CD, Herdman SJ, Whitney SL et al. Vestibular Rehabilitation for Peripheral Vestibular Hypofunction: An Evidence_Based Clinical Practice Guideline: From the American Physical Therapy Association Neurology Section. J Neurol Phys Ther. 2016; 40(2):124-155

I haven’t heard of it

I have heard of it

I have reviewed it or joined a formal discussion about it

I am very familiar with it

Q8 Indicate your agreement with the following:

Vestibular rehabilitation is effective for…

patient with acute (0-2 weeks) and subacute (2 weeks - 3month)vestibular hypofunction

patient with chronic (>3 months) vestibular hypofunction

patient with bilateral vestibular hypofunction

reducing patients’ anxiety and level of disability

Q9. Do you provide vestibular hypofunction patients with customized exercises?

Always

Sometime

Almost never

Q10. On average, how often do you supervise your patients’ with vestibular exercise programs?

Several times per week

Weekly

Every few weeks

Monthly

Q 11. On average, what is the target number of total minutes that you ask your patients with acute/subacute vestibular hypofunction to spend on their exercises daily?

XXX minutes

Q12 On average, what is the target number of totals minutes that you ask your patients with chronic vestibular hypofunction to spend on their exercises daily?

XXX minutes

Q13. On average, how many total times do you see patients with acute/subacute unilateral vestibular hypofunction?

XX times

Q14. On average, how many total times do you see patients with chronic unilateral vestibular hypofunction?

XX times

Q15. On average, how many total times do you see patients with bilateral vestibular hypofunction?

XX times

Q16. Which of the following are reasons that you might stop a course of care for a patient with vestibular hypofunction (Select all that apply)

Achievement of primary goals

Resolution of symptoms

Plateau in progress

Q17. Which, if any, standardized measures do you use regularly for patients with peripheral vestibular hypofunction? Please indicate how often you assess them (e.g. BERG Balance Scale, only at initial eval)

Q18. What are the biggest barriers that you face to providing optimal care to patient with vestibular hypofunction?

(listed/coded)

Patient access to clinic

Patient ability/willingness to do home exercise program

Psych/Soc issues

Medical comorbidities

Equipment

Vestibular suppressants

Insurance restrictions

Missed visits

Coordinating with physicians

Q19. Name one of two important facilitators promoting effective care of patients with vestibular hypofunction in your practice (in other words, what works well)

(listed/coded)

Quality patient education

Connecting with the painter

Clinician support

Setting expectations

Regular visits with progressions

Early intervention

Tracking log

**Supplementary File 2**

**WIDER Reporting Checklist for Knowledge Translation Interventions**

| **WIDER Reporting Standard** | **Site A** | **Site B** | **Site C** | **Site D** | **Site E** |
| --- | --- | --- | --- | --- | --- |
| i. Characteristics of those delivering the intervention (credentials, experience, role in organization) | HR (site lead 2,3,4,6) | SM (site lead 2,3,4,6) | LD (site lead 2,3,4,6) | RH (site lead 2,6) | KS (site lead 2,4,6) |
|  | Credentials: (1) Novice, 2) 5+yrs clinical experience, (3) American Board of Physical Therapy Specialties Certified Clinical Specialist, (4) Expertise in vestibular dysfunction, (5) Resident/Fellow, (6) Academic Faculty | | | | |
| ii. Characteristics of the participants (credentials, experience, role in organization) | - Setting: Hospital-based, outpatient, private - PTs: 11 - Credentials represented: (2) - Mean yrs experience: 6.4yrs | - Setting: Outpatient, private - PTs: 2 - Credentials represented: (2,3,4) - Mean yrs experience: 5.0 | - Setting: Outpatient, private - PTs: 4   Credentials represented: (1, 2, 3, 4)   - Mean yrs experience: 4.5 | - Setting: Hospital-based outpatient, private - PTs: 6 - Credentials represented: (1,2,3,4,5) - Mean yrs experience: 6.6 | - Setting: Hospital-based inpatient and outpatient, government - PTs: 22 - Credentials represented: (1, 2, 3, 4, 5) - Mean yrs experience: 3.4 |
|  | Credentials: (1) Novice, (2) 5+yrs clinical experience, (3) American Board of Physical Therapy Specialties Certified Clinical Specialist, (4) Expertise in vestibular dysfunction, (5) Resident/Fellow, (6) Academic Faculty; PTs=Physical Therapists | | | | |
| iii. Outcome Measures | Percentage of episodes of care or visits with:   1. Handout Offered 2. Videos Offered 3. Text Messages Offered 4. GS Exercise Program Offered 5. GS Dose Recorded 6. Patient Reported Adherence Recorded 7. Patient Reported Minutes Recorded | Percentage of episodes of care or visits with:   1. Exercise Instruction Handout Offered 2. Exercise Log Offered 3. Exercise Targets Offered 4. Timer Offered 5. Text Messages Offered 6. GS Exercise Program Offered 7. GS Dose Recorded 8. Patient Reported Adherence Recorded 9. Patient Reported Minutes Recorded | Percentage of episodes of care or visits with:   1. Educational Handouts Offered 2. Timer Offered 3. Metronome Offered 4. App Offered 5. GS Exercises Prescribed 6. GS Dose Recorded 7. Patient Reported Adherence Recorded 8. Anxiety Addressed | Percentage of episodes of care or visits with:   1. Exercise Instruction Handout Provided 2. Documentation Template Used 3. HEP Practice at Visit 4. GS Prescribed or Advanced 5. Patient Reported Adherence Documented 6. Anxiety Screen 7. Depression Screen | 1. Tally of the number of patients with hypofunction identified by each clinic;  2. Therapist self-efficacy survey scores (confidence in treatment);  3. Therapist participation in training program |
| iv. Mode of delivery  v. The intensity:  **Audit and Feedback** | - Chart review feedback provided verbally in a group setting and individually in written from. Group discussion’s addressed progress and needs to modify intervention - Monthly | - Chart review feedback provided verbally with discussion about progress and needs to modify intervention - Monthly | - Chart review feedback provided verbally with discussion about progress and needs to modify intervention - Monthly | - Chart review feedback provided verbally with discussion about progress and needs to modify intervention - Monthly | **Initially:**. **Follow-up:** Track and Report: 1. How many people are trained, 2. How many people with vestibular hypofunction have been identified via chart review. Monthly meetings to report chart review results and hear stakeholder feedback about needs/barriers. |
| iv. Mode of delivery  v. The intensity:  **Communities of Practice** | - Participating therapists met **monthly** to review audit feedback, discuss implementation efforts, and learn from each other about care for the target population. | - Participating therapists met **monthly** to review audit feedback, discuss implementation efforts, and learn from each other about care for the target population. | - Participating therapists met **monthly** to review audit feedback and discuss implementation efforts. | - Participating therapists met **monthly** to review audit feedback, discuss implementation efforts, and learn from each other about care for the target population. | **Monthly** peer to peer meetings to review clinical practice: case studies were reviewed formally or presented by the study participants to facilitate clinical experiences and develop and mentor interventions within each clinic in addition to open discussion |
| iv. Mode of delivery  **Educational Materials** | - Single Page Guideline Summary Handout (link) | - Single Page Guideline Summary Handout (link) | - Single Page Guideline Summary Handout (link) | - Single Page Guideline Summary Handout (link) | - Single Page Guideline Summary Handout (link) - Self-guided online course (X hours) - Refresher videos available online at on iPads at point of care |
| iv. Mode of delivery  v. The intensity:  **Educational Meetings** | - One educational meeting to teach therapists how to use patient reminder text message system |  |  |  | - In person lecture/lab educational course (8 hours) - Skills competency assessment |
| iv. Mode of delivery  v. The intensity:  **Local Consensus Process** | - Site participants worked together to determine target guideline adherence goals. - Site participants worked together intermittently over 6 months to create:  1. patient education handouts 2. YouTube videos for patient education  - a standardized system for documenting exercise prescriptions and patient reported adherence | - Site participants worked together to determine target guideline adherence goals. - Site participants worked together intermittently over 6 months to:  1. load pictures, videos, and written instructions to establish a customized exercise library. 2. work with a graphic designer to develop a printable home exercise log. 3. organize patient resources for easy accessibility 4. develop laminated cards printed with a focal target (3 different sizes) to be issued to patients for gaze stabilization exercises. 5. create a common documentation template | - Site participants worked together to determine target guideline adherence goals. - Site participants worked together intermittently over 6 months to create:  1. create patient education handouts (three) 2. template phrases to the electronic health records to prompt therapists to collect and report needed patient adherence information 3. organize patient resources for easy accessibility | - Site participants worked together to determine target guideline adherence goals. - Site participants worked together intermittently over 6 months to create:  1. updated and more detailed patient education handouts 2. a documentation template to prompt therapists to collect and report needed patient information 3. a screening tool to identify patients with signs consistent with anxiety and/or depression | - Site participants worked together to determine target guideline adherence goals. |
| iv. Mode of delivery  v. The intensity:  **Local Opinion Leaders** | - Site leader was a recognized expert in vestibular rehabilitation. Held a position of combined day-to-day practice and administrative leadership responsibilities.  1. Site leader was available full time, onsite. | - Site leader was a recognized expert in vestibular rehabilitation practicing daily in the clinical setting.  1. Site leader was available full time, onsite. | - Site leader was a recognized expert in vestibular rehabilitation working at a nearby academic institution.   Site leader was available daily by email/phone. | - Site leader was a recognized expert in vestibular rehabilitation. Held a position of combined day-to-day practice and administrative leadership responsibilities.   Site leader was available full time, onsite. | - Site leader was a recognized expert in vestibular rehabilitation. Held a position of combined day-to-day practice and administrative leadership responsibilities. - Site leader was available full time, onsite. |
| iv. Mode of delivery  v. The intensity:  **Reminders** | - Documentation template prompted therapists to collect and report targeted patient information. - Available for each patient visit during and after intervention. | - Documentation template prompted therapists to collect and report targeted patient information. - Available for each patient visit during and after intervention. | - Documentation template prompted therapists to collect and report targeted patient information. - Available for each patient visit during and after intervention. | - Documentation template prompted therapists to collect and report targeted patient information. - Available for each patient visit during and after intervention. |  |
| iv. Mode of delivery  v. The intensity:  **Resources provided to therapists to offer to patients** | - Educational handout - YouTube video links - Text message reminder system | - Educational handout - Text message reminder system - Timers - Laminated GS cards - Metronomes | - Handouts (Three – targeted toward beginning, middle, end of treatment) - Timers - Metronomes - Interactive communication app (available pre-study) - Reference list of care providers for patients with anxiety. | - Educational handout | - Patient education videos on facility video resource network. |
| vi. Intervention duration | - 6 months | - 6 months | - 6 months | 6 months | - 6 months |
| vii. Adherence/fidelity to delivery protocols (if you plan to meet monthly, do you?) | Bi-weekly site leader meetings were held monthly with study PI and other team members to review progress, adherence, and fidelity and to find solutions to challenges.  Fidelity challenges: Text message system was not available until month 4th of the 6-month intervention. | Bi-weekly site leader meetings with monthly reporting and discussion - specific focus on chart review completion, sharing of results with stakeholders, responding to stakeholder meetings | Bi-weekly site leader meetings with monthly reporting and discussion - specific focus on chart review completion, sharing of results with stakeholders, responding to stakeholder meetings  The plan to increase use of a patient communication app was discontinued mid intervention due to lack of interest.  The plan for monthly meetings with participating therapists was changed to every other month because therapists did not have a common time when they could meet (staggered lunch schedules). | Bi-weekly site leader meetings with monthly reporting and discussion - specific focus on chart review completion, sharing of results with stakeholders, responding to stakeholder meetings.  A plan to train therapists and implement a patient engagement tool (communication app) to assist with patient education and adherence was discontinued due to insufficient time to develop it. | Bi-weekly site leader meetings with monthly reporting and discussion - specific focus on monthly data collection, sharing of results with stakeholders, responding to stakeholder meetings |
| viii. How the intervention was developed | Stakeholder meetings regarding target CPG action statements and focus on best available evidence to promote behavior change among therapists and exercise adherence among patients | Stakeholder meetings regarding target CPG action statements and focus on best available evidence to promote behavior change among therapists and exercise adherence among patients | Stakeholder meetings regarding target CPG action statements and focus on best available evidence to promote behavior change among therapists and exercise adherence among patients | Stakeholder meetings regarding target CPG action statements and focus on best available evidence to promote behavior change among therapists and exercise adherence among patients | Stakeholder meetings regarding target CPG and focus on therapist needs with regard to meeting needs of patients with vestibular hypofunction. Identified need for training on how to identify and initiate treatment for persons with vestibular hypofunction. |
| xi. Mechanisms used to change the intervention as needed | Updated educational information based on stakeholder feedback, text message updates | Based on meetings with PTs, supplemental meetings with patients later in the intervention | Based on meetings with PTs, supplemental meetings with patients later in the intervention | Based on meetings with PTs, supplemental meetings with patients later in the intervention | Based on meetings with PTs, supplemental meetings with patients later in the intervention |
| x. What the intervention was aiming to change | Improve therapist behaviors to better align with guideline Action Statement 5, 7: Provide targeted exercises and promote optimal dose for home exercise program | Improve therapist behaviors to better align with guideline Action Statement 5, 7: Provide targeted exercises and promote optimal dose for home exercise program | Improve therapist behaviors to better align with guideline Action Statement 5, 7: Provide targeted exercises and increase compliance with home exercise program. | Improve therapist behaviors to better align with guideline Action Statement 5, 7: Provide targeted exercises and promote optimal dose for home exercise program | Improve therapist behaviors to better align with guideline Action Statement 1-3 and 9: Identifying people who should be offered VR. Awareness of factors that impact outcomes. |

**Supplementary File 3.**

**Detailed Description of Site Interventions**

*Site A.*

Seven target behaviors were developed by site participants. Three behaviors involved therapists providing patients with resources to support exercise adherence. Four behaviors were designed to increase therapist attention to intentionally prescribing, collecting, and documenting their patients’ home exercise program and the patients’ reported adherence to the program.

1) Patient Education Handout: Site participants developed a patient-centered educational handout to improve patients’ knowledge of their condition and the importance of exercise program adherence. The implementation goal was for each patient to receive this handout during their episode of care.

2) Demonstration YouTube Videos: Site participants developed educational videos demonstrating correct techniques for patients to conduct gaze stabilization exercises. The implementation goal was for each patient to receive a copy of the link to these videos during their episode of care.

3) Text Message Reminders: The site established a contract with a company to provide text message reminders through an online, HIPAA compliant platform. Text message reminders to patients were sent three times a day at designated times, stating: “Time to do your exercises! Keep up the good work!”. The implementation goal was for each patient to be invited to receive reminder text messages.

4-7) Visit Record Restructure: Participants created a standardized system for documenting exercise prescriptions and patient reported adherence. The implementation goal was for therapists to document at each visit: 4) prescribed exercises, 5) prescribed dose,6) the patient’s report regarding overall exercise adherence, 7) and of daily minutes of home exercises completed.

*Site B.*

Nine target behaviors were developed by site participants. Five behaviors involved therapists providing patients with resources to support exercise adherence. Four additional behaviors were designed to increase therapist attention to collecting and documenting their patients’ exercise adherence.

1) Patient Exercise Handouts: An exercise software platform was purchased that allowed patients to be provided with fully customized exercises including photographs and videos. The participants loaded pictures, videos, and written instructions to establish a customized exercise library. The implementation goal was for each patient to receive these handouts during their episode of care.

2) Exercise Log Handout: Participants worked with a graphic designer to develop a printable home exercise log. The implementation goal was for each patient to be offered an exercise log during the episode of care.

3) Timers: Simple timers were purchased from discount stores to support patients’ ability to accurately time their gaze stability exercises. Timers were organized on an easily accessible shelf in the therapist work area. The implementation goal was for each patient to be offered a timer during their episode of care.

4) Gaze Stabilization Exercise Targets : Laminated cards printed with a focal target (3 different sizes) were developed by the participants to be issued to patients for gaze stabilization exercises. Exercise targets were organized on an easily accessible shelf in the therapist work area. The implementation goal was for each patient to be offered exercise targets during their episode of care.

5) Text Message Reminders: Participants identified a text messaging system that allowed for customized text message reminders. A sampling of text messages was compiled including simple messages “a friendly reminder to do your exercises today.” The implementation goal was for each patient to be invited to receive reminder text messages.

6-9) Visit Record Restructure: Specific pre-established phrases were added at the beginning and end of each patient encounter entry in the site’s electronic medical record. These ‘smart phrases’ prompted the participants to record specific information. The implementation goal was for therapists to document at each visit: 6) prescribed exercises, 7) prescribed dose, 8) the patient’s report regarding exercise adherence, and 9) patient report of daily minutes of home exercises completed.

*Site C.*

Eight target behaviors were developed with site participants. Four behaviors involved therapists providing patients with resources to support exercise adherence. Three behaviors were designed to increase therapist attention to collecting and documenting their patients’ exercise adherence. One behavior involved providing referral information for patients presenting with symptoms consistent with anxiety.

1) Educational Patient Handouts: Three informational handouts for patients were developed to meet patient education needs at three distinct timepoints, ‘initial evaluation’, ‘mid therapy’ and at the ‘end of therapy’. The implementation goal was for therapists to provide patients handouts at the indicated time point of their course of care.

2-3) Timers and Metronomes: These items were purchased for participants to share with patients who needed to time and monitor cadence of their exercise program. Participants were also exposed to a smartphone metronome app that they could share with patients. The implementation goal was for each patient to be offered available tools to support exercise compliance during their episode of care.

4) Smart phone application: Participants were encouraged to engage patients in use of an already in-use smartphone app to monitor patient exercise adherence. The app included a messaging feature between patient and therapist. The implementation goal was for therapists to offer patients to use the smart phone app to track exercises and communicate with their therapist as needed.

5-7) Visit Record Restructure: The sites’ electronic medical record system was modified by adding specific pre-established ‘smart phrases’. These ‘smart phrases’ prompted the participants to record specific information. The implementation goal was for therapists to document 5) prescribed exercises, 6) exercise dose, and 7) to document patients’ report of home exercise adherence for each visit.

8) Referral list for patients with anxiety: Participants were provided with a list of local area psychologists with expertise in anxiety. The implementation goal was for therapists to provide patients identified with symptoms consistent with anxiety with the referral list.

*Site D.*

Eight target behaviors were planned with site participants. Two behaviors involved therapists providing patients with a resource to support exercise adherence. Four behaviors were designed to increase therapist attention to collecting and documenting their patients’ exercise adherence. Two behaviors involved screening patients for symptoms consistent with depression and anxiety.

1) Patient Exercise Handouts: Site participants updated their home exercise instruction materials with improved detail related to dosage and frequency and images. The implementation goal was for each patient to receive these handouts during their episode of care.

2) Electronic Communication Application: Site participants planned to design and implement an electronic communication application to provide reminders to exercise and support to patients. The implementation goal was to offer this application to patients during their episode of care once it was developed.

3-6) Visit Record Restructure: A standardized documentation template was created within the electronic record to prompt therapists to consistently address specific elements of care to promote exercise adherence, including details about patients’ prescribed home exercise programs and how therapists progressed the program. The implementation goal was for therapists to 3) use the documentation template and within that to 4) record the home exercise program prescribed, 5) whether the exercise program was practiced during the session, and 6) patient reported adherence to the exercise program.

7-8) Screening and Referral for Anxiety and Depression: Therapists developed a screening tool to identify patients with signs consistent with anxiety and/or depression. The implementation goal was for therapists to screen for anxiety(Spitzer RL 2006) and depression(Kroenke K 2003) during each patients’ episode of care.

*Site E.*

Five interventions were identified by study participants and developed by expert clinicians and the hospitals’ medical media team. All five interventions were designed to improve therapist self-efficacy for assessing patients for vestibular dysfunction and their likelihood to assess patients for vestibular dysfunction, and offer rehabilitation. The implementation goals for all interventions were 1) to increase participant self-efficacy for assessing patients for vestibular dysfunction and 2) to increase the frequency that therapists conducted assessments of patients to identify possible vestibular dysfunction.

1-2) Therapist Training: Participants were invited to participate in one or two educational courses. The implementation goal was for participants to experience an increase in self-efficacy for assessing and treating patients with peripheral vestibular hypofunction.

- 1. In-person Education Course: Participants were invited to participate in an in-person eight hour lab/lecture course.The course included basic anatomy, physiology of the vestibular system; learning hands-on skills to assess for persons with dizziness, case studies to develop differential diagnosis, and treatment/interventions. The case studies surrounded and included the action statements from the CPG. The implementation goal was for participants to complete the education course and for participants to experience an increase in self-efficacy for assessing and treating patients with peripheral vestibular hypofunction.
  2. Self-guided Therapist Education Course: Participants were provided with self-guided optional and enduring training resources regarding assessment for vestibular dysfunction that was provided on a Veteran’s Administration internal network that mimicked the in-person training session.

3) Therapist Enduring Education Resources: Participants were provided with resources to aid in continued education and ‘refresher’ videos. These ‘how to’ video resources were available on the VA internal network, the VA external network and uploaded to iPADs provided to 4 participating clinics. The videos were closed captioned to allow the clinician to access the video and instructions while performing an assessment or treatment, with or without sound. The implementation goal was for participants to use these resources and to experience an increase in self-efficacy for assessing and treating peripheral vestibular hypofunction.

4) Skills Competency Assessment: Participants were invited to complete a competency assessment at the completion of the in-person or self guided education course. The implementation goal was for participants to complete and pass the skills competency assessment.

5) Ongoing In-person Education: Participants were provided with monthly peer to peer meetings to review clinical practice, open clinic discussions and case studies were reviewed formally or presented by the study participants to facilitate clinical experiences and develop and mentor interventions within each clinic. The implementation goal was for participants to participate in these sessions.

**Supplementary File 4.**

**Consolidated Framework for Implementation Research-Informed and Organizational Readiness to Implement Change**

**Pre and Post Survey Results**

| **Item** | **Site A** | **Site B** | **Site C** | **Site D** | **Site E** |
| --- | --- | --- | --- | --- | --- |
|  | **Percentage of Participants Reporting Agree or Strongly Agree** | | | | |
| Respondents (n) Pre/Post | 11/8 | 2/2 | 4/3 | 4/3 | 22/17 |
| **CFIR Informed Item - Positive Agreement** | Pre %/Post % | Pre %/Post % | Pre %/Post % | Pre %/Post % | Pre %/Post % |
| 1. The individual(s) who developed this intervention understand my practice setting | 91/88 | 100/100 | 100/100 | 100/100 | 100/100 |
| 1. This intervention is important compared to other interventions our facility could be implementing | 100/100 | 100/100 | 100/100 | 100/100 | 86/94 |
| 1. This intervention fits with our patients' needs, preferences, and resources | 100/100 | 100/100 | 100/100 | 100/100 | 100/100 |
| 1. This intervention is in alignment with external incentives and pressures our facility is dealing with | 82/83 | 100/100 | 100/67 | 50/67 | 64/93 |
| 1. The culture in our facility supports success of initiatives like this intervention | 91/75 | 100/100 | 100/100 | 100/100 | 96/100 |
| 1. There is a strong need for this intervention at our facility | 100/88 | 100/100 | 50/33 | 100/100 | 91/100 |
| 1. It will be difficult to fit this intervention into our existing workflow *(disagree/strongly disagree responses)* | 91/57 | 100/100 | 100/67 | 100/67 | 86/76 |
| 1. The goals of this intervention are clear | 100/100 | 100/100 | 100/67 | 100/100 | 77/82 |
| 1. There is a plan to provide regular feedback on how well we are implementing this intervention | 82/88 | 100/100 | 100/67 | 100/100 | 77/82 |
| 1. We have sufficient resources to implement this intervention | 73/88 | 100/100 | 100/100 | 100/100 | 86/100 |
| 1. I have the information I need to implement this intervention | 91/100 | 100/100 | 100/100 | 100/33 | 82/100 |
| 1. I feel positively about this intervention | 100/100 | 100/100 | 100/100 | 100/100 | 100/100 |
| 1. I feel confident in our facility's ability to implement this intervention | 100/88 | 100/100 | 100/100 | 100/100 | 100/100 |
| 1. I'm prepared to carry out this intervention | 100/100 | 100/100 | 100/100 | 100/100 | 91/100 |
| **ORIC Item** |  |  |  |  |  |
| 1. People who work here are committed to implementing this change (C1) | 91/88 | 100/100 | 100/100 | 100/100 | 86/94 |
| 1. People who work here feel confident that they can keep track of progress in implementing this change | 82/75 | 100/100 | 100/100 | 100/100 | 95/100 |
| 1. People who work here will do whatever it takes to implement this change | 55/63 | 100/100 | 100/100 | 100/67 | 77/88 |
| 1. People who work here feel confident that the organization can support people as they adjust to this change | 73/63 | 100/100 | 100/100 | 100/67 | 95/100 |
| 1. People who work here want to implement this change | 82/100 | 100/100 | 75/67 | 100/100 | 86/100 |
| 1. People who work here feel confident that they can handle the challenges that might arise in implementing this change | 82/88 | 100/100 | 100/100 | 100/67 | 86/35 |
| 1. People who work here are determined to implement this change | 80/75 | 100/100 | 75/100 | 75/33 | 86/100 |
| 1. People who work here feel confident that they can coordinate tasks so that implementation goes smoothly | 91/63 | 100/100 | 100/100 | 100/100 | 91/94 |
| 1. People who work here are motivated to implement this change | 82/88 | 100/100 | 100/100 | 100/67 | 91/94 |
| 1. People who work here feel confident that they can manage the politics of implementing this change | 64/63 | 100/100 | 100/100 | 75/100 | 91/94 |
